# Late Onset of CSF Rhinorrhea in a Postoperative Transsphenoidal Surgery Patient Following Robotic-Assisted Abdominal Hysterectomy

**DOI:** 10.1177/2324709614520982

**Published:** 2014-01-24

**Authors:** Justin T. Dowdy, Marcus W. Moody, Christopher P. Cifarelli

**Affiliations:** 1 Department of Neurosurgery, University of Arkansas for Medical Sciences, Little Rock, AR, USA; 2 Department of Head & Neck Oncology, University of Arkansas for Medical Sciences, Little Rock, AR, USA

**Keywords:** CSF rhinorrhea, transsphenoidal, pituitary, CSF leak, robotic-assisted surgery

## Abstract

Cerebrospinal fluid (CSF) leak is the most commonly encountered perioperative complication in transsphenoidal surgery for pituitary lesions. Direct closure with a combination of autologous fat, local bone, and/or synthetic grafts remains the standard of care for leaks encountered at the time of surgery as well as postoperatively. The development of the vascularized nasoseptal flap as a closure technique has increased the surgeon’s capacity to correct even larger openings in the dura of the sella as well as widely exposed anterior skull base defects. Yet these advances in the technical nuances for management of post-transsphenoidal CSF leak are useless without the ability to recognize a CSF leak by physical examination, clinical history, biochemical testing, or radiographic assessment. Here, we report a case of a patient who developed a CSF leak 28 years after transsphenoidal surgery, precipitated by a robotic-assisted hysterectomy during which increased intra-abdominal pressure and steep Trendelenberg positioning were both factors. Given the remote nature of the patient’s transsphenoidal surgery and relative paucity of data regarding such a complication, the condition went unrecognized for several months. We review the available literature regarding risk and pathophysiology of CSF leak following abdominal surgery and propose the need for increased vigilance in identification of such occurrences with the increasing acceptance and popularity of minimally invasive abdominal and pelvic surgeries as standards in the field.

## Introduction

Cerebrospinal fluid (CSF) rhinorrhea is a well-documented complication of transsphenoidal surgery (TSS) for pituitary and parasellar lesions.^1-11^ The risk of postoperative CSF leak ranges from 2.3% to 13% in larger series,^1,2,8,9^ with advanced technical experience of the surgeon further reducing the incidence to approximately 2%.^2^ Associated sequelae of untreated CSF leaks may include tension pneumocephaly, prolonged hospitalization, and meningitis, which may be life-threatening.^1,2,5,9^ Both repeat transsphenoidal surgery (RTSS) for recurrent disease and the occurrence of CSF flow within the surgical field requiring immediate surgical repair are independent factors for the development of subsequent CSF rhinorrhea.^5-8^ Such leaks commonly present within 1 week of initial surgery; however, CSF rhinorrhea has been reported to occur up to 10 years after TSS.^6,11^

In this report, we describe the case of a patient who developed a CSF leak and subsequent meningitis after undergoing a robotic-assisted hysterectomy. Interestingly, the CSF leak developed in the immediate postoperative period following the robotic procedure, separated by 28 years from the initial TSS, prompting our review of potential pathophysiological mechanisms by which such a CSF leak could occur.

## Case Illustration

**Figure 1.**
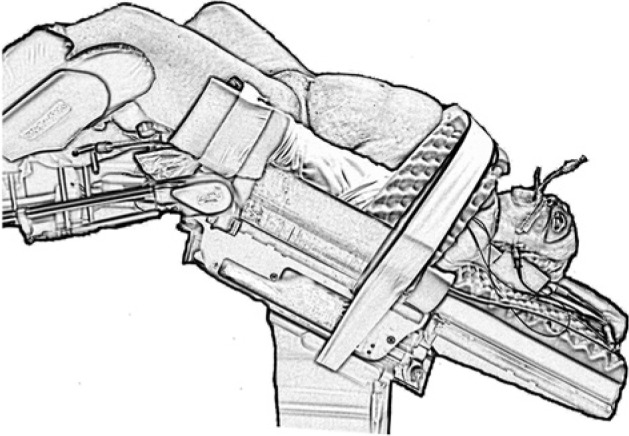
Schematic representation of the steep Trendelenberg position with the patient’s head position approximately 45° down.

**Figure 2.**
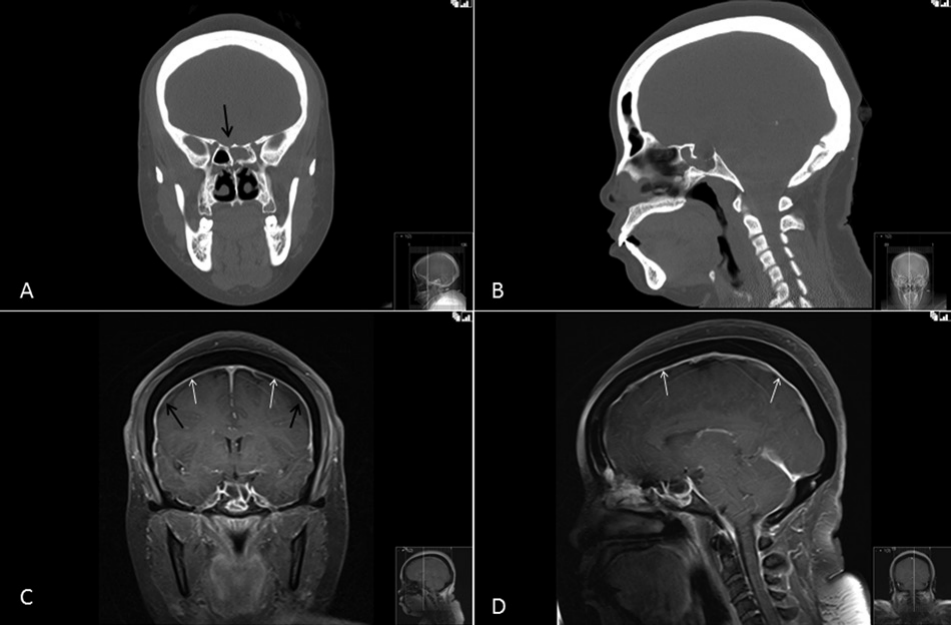
Coronal (A) and sagittal (B) views of the maxillofacial computed tomography prior to endoscopic repair show a small defect in the floor of the sella (arrow, A) and postoperative changes from the original transsphenoidal surgery. Coronal (C) and sagittal (D) post-contrast magnetic resonance images illustrate thick dural enhancement (white arrows, C and D) and the presence of subdural hygromas (black arrows, C) suggestive of intracranial hypotension.

**Figure 3.**
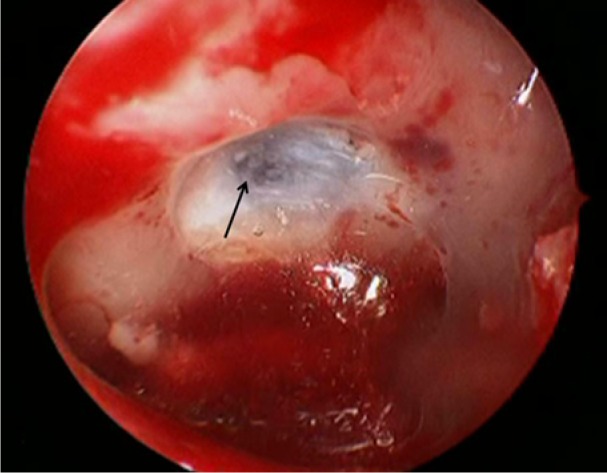
Intraoperative photograph demonstrating the defect at the floor of the sella turcica (arrow) with active extravasation of cerebrospinal fluid.

**Figure 4.**
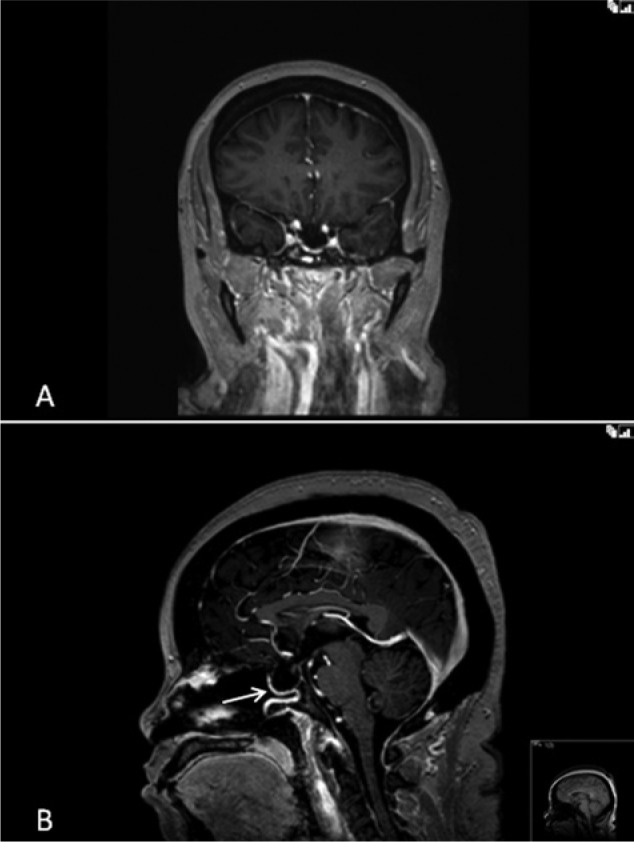
Coronal (A) and sagittal (B) post-contrast magnetic resonance images 16 months after the cerebrospinal fluid (CSF) leak repair show resolution of the dural enhancement and subdural hygromas seen in Figure 1 (C-D). Note the presence of a vascularized septal flap (arrow, B) and return of the brainstem and cerebellum to a normal anatomic position in comparison to the inferior displacement noted in Figure 1 (D). These findings indicate full resolution of intracranial hypotension caused by the CSF leak.

The patient is a woman who underwent a sublabial transsphenoidal approach for resection of a pituitary adenoma at the age of 30. An autologous abdominal fat graft was applied at the sellar floor secondary to intraoperative observation of a CSF leak. She tolerated the procedure well and had an uneventful postoperative course. The pathology of the lesion was consistent with a nonfunctional pituitary macroadenoma. At the age of 58, the patient underwent a robotic-assisted total hysterectomy and oophorectomy due to persistent menorrhagia. During the procedure, the operative table was placed in extreme Trendelenberg position, noted to be approximately 45° from the floor (Figure 1). In the immediate postoperative period, the patient developed a clear nasal discharge that could be provoked with positional change. The working diagnosis for this postoperative drainage was allergic rhinitis versus an upper respiratory infection. Three months later, the patient returned to a local emergency department with severe headache, photophobia, nuchal rigidity, and continued positional nasal discharge. The nasal drainage was collected and tested positive for β-2 transferrin, confirming the presence of an active CSF fistula. Magnetic resonance imaging and computed tomography scan of the brain revealed a 2 to 3 mm defect located in the mid-floor of the sella with diffuse dural enhancement and bilateral subdural hygromas (Figure 2A-D). The patient underwent an endoscopic endonasal approach to the sphenoid sinus and sellar region. After bony decompression of the residual sphenoid rostrum, the mucosal elements at the sellar floor were removed via sharp dissection. A defect in the previous sellar floor reconstruction was easily identified with active extravasation of CSF (Figure 3). Reconstruction of the water-tight layer was accomplished by using a combination of autologous bone from the posterior nasal septum, a vascularized nasal septal flap, and a 1-square-inch piece of Duragen artificial dural implant (Integra LifeSciences, Plainsboro, NJ). The patient tolerated the procedure well and was able to be discharged home 2 days later on a course of intravenous antibiotics for meningitis. The patient was evaluated at a routine clinic visit 2 weeks after repair, where no further CSF rhinorrhea was reported or observed. Follow-up imaging at 16 months postoperatively demonstrated an intact vascularized closure and no evidence of persistent CSF leak (Figure 4A and B).

## Traditional CSF Rhinorrhea Etiology and Management

The causes of CSF fistulae of the anterior skull base are varied, ranging from infection to tumor invasion and, quite commonly, surgical/intraoperative leak. The majority of the latter are immediately recognized by the surgical team and repaired via autologous fat graft, artificial dural substitute, vascularized nasoseptal flap, or any combination of the aforementioned. More insidious CSF fistulae caused by infection or tumor are also managed in the same manner, usually using an endoscopic endonasal approach for identification and repair, albeit more delayed in initial diagnosis than a witnessed intraoperative CSF leak. Adjuncts to treatment such as intrathecal injection of fluorescent dyes or placement of a lumbar drain aid in both identification and repair of occult CSF leaks where defects in the anterior skull base cannot be clearly seen on radiographic imaging.^12,13^

Traumatic injury to the anterior skull base resulting in CSF rhinorrhea complicates approximately 2% to 3% of all head trauma cases.^14,15^ Unlike postsurgical CSF leaks, the treatment of traumatic CSF leaks associated with anterior skull base fractures can range from immediate surgical repair to conservative management, as the rate of spontaneous resolution can be up to 60% of cases with conservative management including placement of a lumbar drain for temporary CSF diversion.^12,16^ Larger defects in the skull base consistently require surgical repair, using an endoscopic or open approach.^17,18^

## Intracranial Pressure During Minimally Invasive Abdominal-Pelvic Surgery

The advancement of minimally invasive approaches to intra-abdominal and pelvic surgeries has been dependent on the development of improved endoscopic tools as well as optimization of patient preparation, including anesthetics and positioning. While pneumoperitoneum creates the necessary working space for the placement of surgical instrumentation, patient positioning allows for passive manipulation of viscera out of the surgical field, often requiring extreme angulation of the operating table. Steep Trendelenberg, usually defined as a 45° angle from the horizontal plane, is considered a standard position for many robotic and laparoscopic surgeries, including minimally invasive gynecologic and urologic procedures, such as hysterectomies and prostatectomies.^19-21^ Unfortunately, the resultant dependencies of the head and neck often manifest in postoperative facial edema, with the risk being higher in cases with longer operative times.^22,23^ Similarly, the risk of increased intraocular pressure (IOP) makes such positioning, and the procedures requiring such position, a relative contraindication for patients with previously diagnosed glaucoma.^24^ Studies of the direct measurement of IOP under steep Trendelenberg and pneumoperitoneum have shown a reversible increase in IOP, with operating room table angulation having a greater impact on the degree of IOP increase than the degree of pneumoperitoneum.^25^ Although IOP is not a reliable predictor of intracranial pressure (ICP), the venous hypertension caused by the head down position leading to increased IOP also serves to increase the intracranial pressure.^26,27^ Such increases in ICP have a clear association with the development of a spontaneous CSF fistula as well as compromise of a preexisting skull base defect.^28^

Even in the absence of steep Trendelenberg, pneumoperitoneum can cause an independent increase in intracranial pressure. In cases of intra-abdominal hypertension (IAH), ICP has been found to be elevated.^26^ Intraoperative measurements of ICP in multisystem trauma patients undergoing laparoscopic procedures without significant Trendelenberg can result in an increase in ICP ranging from 9 to 60 mm Hg within 10 minutes.^29^ In patients with ventriculoperitoneal shunts for hydrocephalus who undergo a subsequent laproscopic procedure, evaluation of the ICP revealed an increase during maximal insufflation. Recommendations from this work include that special attention be given to shunted patients with an intraperitoneal catheter during laparoscopic surgery, although several groups have shown such use of laparoscopy without neurological injury.^30-33^ Additional data from large animal studies have confirmed the relationship between increased abdominal pressure and the resulting increase in intracranial pressure during pneumoperitoneum.^34-36^

## Durability of CSF Leak Repair

Improvements in graft material and the development of dural sealants have advanced the ability to definitively repair a CSF fistula, regardless of its etiology. Several retrospective studies have determined that repairs with such material remain viable indefinitely.^37,38^ The normal mucosal regrowth that occurs following such repairs is believed to create permanent barrier across the defect, hence the importance of the presence of healthy mucosa within the sinuses during the healing process.^39,40^ Hadad et al^41^ described the early use of the local nasoseptal flap to cover defects in the skull base and prevent CSF fistula progression. In this setting, the vascular supply from the tissue pedicle alleviates need for mucosal in-growth across a defect. Yet, even in the setting of a vascularized flap closure, a repair of the CSF fistula may fail, usually due to persistently increased intracranial pressure, requiring the need for CSF shunting.^42^ No clinical data exist regarding transient increases in ICP and recurrence of CSF leak. Yet, despite this fact, prolonged increases in ICP, that is, over 1 to 3 hours, as would be expected during steep Trendelenberg and pneumoperitoneum, could reasonably result in adequate disruption of a “healed” CSF fistula with resultant postoperative CSF rhinorrhea as demonstrated by the current clinical case.

## Conclusions

Laparoscopic and robotic-assisted abdominal and pelvic surgeries have been shown to be safe and effective approaches for abdominal and pelvic surgeries based on both perioperative and short-term outcomes, although long-term outcome studies are still pending.^43-45^ Both steep Trendelenberg and pneumoperitoneum play pivotal roles in the technical execution of such procedures, with the additional risk of a transient increase in intracranial pressure, which, in the absence of a preexistent skull base defect, may not be clinically significant. Patients with known surgical histories including transsphenoidal and anterior skull base procedures should be considered at relatively increased risk for developing a postoperative CSF fistula following the application of steep Trendelenberg and/or pneumoperitoneum. In this patient population, reduction in Trendelenberg angle, minimization of pneumoperitoneum, or exploration of alternative surgical approaches may be of benefit to the patient.
